# Clinical Insights and Radiological Features on Multiple Sclerosis Comorbid with Migraine

**DOI:** 10.3390/jcm14020561

**Published:** 2025-01-16

**Authors:** Maddalena Sparaco, Simona Bonavita

**Affiliations:** 1 2nd Division of Neurology, University Hospital of Campania Luigi Vanvitelli, Via Sergio Pansini, 5, 80131 Naples, Italy; 2 Department of Advanced Medical and Surgical Sciences, University of Campania Luigi Vanvitelli, Piazza Miraglia 2, 80138 Naples, Italy; simona.bonavita@unicampania.it

**Keywords:** multiple sclerosis, migraine, headache

## Abstract

**Background:** Multiple sclerosis (MS) and migraine are neurological diseases, affecting young women. Migraine is the most prevalent type of headache in people with MS (pwMS). **Objectives:** The aim of this review is to describe the clinical, radiological, and therapeutic features of MS and migraine comorbidity. The clinical section focuses on the characteristics of migraine in pwMS and of MS in co-occurrence with migraine, and on the presence of other possible comorbidities. The radiological section deals with the differential diagnosis of white matter lesions and changes in connectivity patterns on brain magnetic resonanceto investigate a possible link between MS and migraine. The therapeutic section evaluates the effects of MS-disease-modifying therapies on migraine and of prophylactic migraine treatments on MS. **Methods:** The literature search was conducted using PubMed as an electronic database. The papers that reported relevant clinical, radiological and therapeutic findings were selected. **Results:** Among 1351 results retrieved, at the end of screening procedures, 34 studies were selected. Migraine can impact the perception of some symptoms and the presence of some comorbidities, particularly relevant in MS. Furthermore, migraine and MS share some radiological features, leading to diagnostic challenges, however identifying some lesion characteristics and changes in the connectivity pathway may be supportive. Medications for migraine and MS should be administered considering both the adverse events and multiple drug interactions. **Conclusions:** The data emerging from this review illustrate the research efforts aimed at providing valuable insights into accurate diagnosis, effective clinical management, and the definition of targeted treatment schedules that could improve the quality of life for pwMS with migraine.

## 1. Introduction

Multiple sclerosis (MS) is a multifactorial autoimmune disease of the central nervous system (CNS), causing demyelination and axonal loss [1].

Another neurological disease more common in women than in men and with a young age of onset is migraine. According to the third edition of the International Classification of Headache Disorders (ICHD-3), migraine is defined as a “recurrent headache disorder manifesting in attacks lasting 4–72 h. Typical characteristics of the headache are unilateral location, pulsating quality, moderate or severe intensity, aggravation by routine physical activity, and association with nausea and/or photophobia and phonophobia” [2].

Recently, a systematic analysis estimated the global, regional, and national burden of disorders that affect the nervous system from 1990 to 2021 [3]. Migraine and MS were both included in the study; in particular, the global ranking of age-standardized DALY rates was 3 for migraine and 26 for MS. Since a comorbidity correction was used to calculate the total prevalence of all nervous system conditions and disorders combined, we do not have a ranking of the global burden for MS when comorbid with migraine.

Interestingly, research has shown a significant comorbidity between MS and migraine. Migraine is the most prevalent type of headache in people with MS (pwMS). A meta-analysis revealed a prevalence of 55% of primary headaches among pwMS [4]. In particular, in the subgroup analysis, the prevalence of migraine was 30%, higher than tension-type headache (22%) [4]. Moreover, migraine is more frequent in pwMS than in the general population. A meta-analysis of 35 studies, published in 2023, involving 279,620 pwMS and 279,603 healthy controls, highlighted that approximately 24% of pwMS experienced migraine, and that pwMS had 1.96-fold increased odds of having migraine compared to healthy individuals [5].

Evaluating the geographical distribution, although with different percentages, the prevalence remains significant across the continents, and the pooled prevalence is 24% in Asia, 25% in Europe, 43% in Africa, and 43% in America [6]. In the USA, the frequency of migraine in pwMS is more than threefold higher both in men (18.4 vs. 5.6%, *p* < 0.001) and women (55.7 vs. 17.1, *p* < 0.001), compared with data from the American Migraine Prevalence and Prevention (AMPP) study (a large epidemiological study of migraine in the US population) [6]. Stratifying by age (20 to over 50), in MS women this result has been confirmed in each age group [7].

Among pwMS with headaches, female sex and young age are overrepresented compared to pwMS without headaches [8,9].

To research a genetic instrumental variable associated with migraine and MS, Horton et al. used a two-sample Mendelian randomization (accessing summary statistics from large genome-wide association studies). Although they did not find any evidence supporting migraine as a causal risk factor for MS, they identified four major histocompatibility complex (MHC) loci (genes HCG20, HLA-B, MSH5, TNXA, and TNXB) increasing the risk for both MS and migraine [10].

By summarizing the current knowledge on the comorbidity of migraine and MS, this review aims to describe the clinical, radiological, and therapeutic features of comorbidity between MS and migraine. Understanding these aspects can help in highlighting the clinical issues, implementing integrated care strategies, and offering a targeted diagnostic pathway that simultaneously addresses both conditions and tailors management strategies accordingly.

## 2. Materials and Methods

The first step consisted of outlining areas of interest in comorbidity between MS and migraine: the selected areas were clinical, radiological, and therapeutic.

In particular, the clinical section aims to identify the characteristics of migraine in pwMS, the features of MS in co-occurrence with migraine, and the presence of other possible comorbidities.

The radiological section deals with the differential diagnosis of hyperintense T2 white matter lesions on brain magnetic resonance imaging (MRI) and reports about lesions location and changes in brain connectivity patterns to investigate a possible link between MS and migraine.

The therapeutic section evaluates the effects of MS-disease-modifying therapies on migraine and the impact of prophylactic migraine treatments on MS.

To ensure a comprehensive and thorough review, the literature search was conducted using PubMed/Medline as an electronic database. Papers were identified using a combination of the following Medical Subject Headings (MeSH) terms: “Multiple Sclerosis” and “migraine” and “clinical” or “radiological” or “MRI” or “therapy” or “treatment”, “disease modifying treatment”, “amitriptyline”,” beta blockers”, “calcium channel blockers”, “topiramate”, “Botulinum Toxin”, “anti-CGRP”. Two independent reviewers evaluated the studies through a two-stage process: an initial screening of titles and abstracts, a subsequent full-text evaluation based on areas of interest and the following inclusion and exclusion criteria. 

### 2.1. Inclusion Criteria

Only studies published in English were considered for inclusion. No date restrictions were applied. Only papers that reported relevant clinical outcomes, radiological findings, and therapeutic options were included. In particular, studies that reported clinical data about migraine or MS through validated questionnaires or measures or clinical interviews were included. Studies including radiological imaging data that show correlations between MS and migraine characteristics were considered. No restrictions were placed on the type of MRI scanner used. Only treatments with approved indications for prophylaxis of migraine and as MS-disease-modifying therapies (DMTs) were considered.

### 2.2. Exclusion Criteria

Papers focused exclusively on MS or migraine or lacking relevant data that do not contribute to an effective understanding of comorbidity were excluded.

Case series, letters without original data, and duplicate studies were excluded.

Articles in languages other than English were excluded. The use of non-validated questionnaires or measures or non-clinically valid interviews or treatments without approved indications for migraine and MS was not considered.

## 3. Results

**Figure 1:**
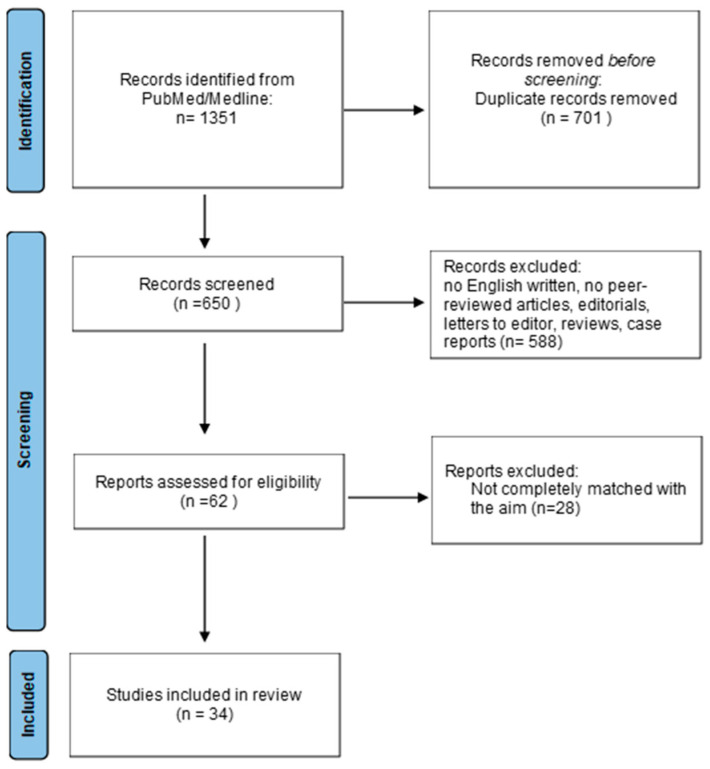
PRISMA flow diagram.

A total of 1351 results were retrieved. Duplicates were removed using Endnote (online version) and manually. All articles that did not match the title profile were discarded, and articles that did not cover topics of interest in both diseases were excluded. At the end of these screening procedures, 34 studies published between 1989 and 2024 that met the objectives of this review were selected and reported (Figure 1).

### 3.1. Clinical Aspects in People with MS and Migraine

#### 3.1.1. Characteristics of Migraine

Evaluating the different types of migraine in MS, the prevalence of migraine without aura was similar across studies, while it was different for migraine with aura. Kister et al. revealed that 36% of pwMS with migraine had auras, in particular visual auras in 44.1% of them, visual/sensory in 38.2%, and only sensory in 17.6% [7]. Instead, Doi et al. found that 5 out of a sample of 127 pwMS had migraine with aura (3.9%) and 21 without aura (16.5%) [11]. Beckman et al. reported that, out of a sample of 754 pwMS, 52 (6.9%) had migraine with aura, 128 (17%) without aura, and 17 (2.3%) had probable migraine without aura [12]. D’Amico et al. among 116 pwMS found no case of migraine with aura and 19 cases of migraine without aura (16.3%) [13]. Möhrke et al., among 98 pwMS, reported headache and in particular 16 with migraine: migraine with aura in 2 (2%) and migraine without aura in 14 patients (14.3%) [8]. 

In the study by Fragoroso et al., 68.3% pwMS with migraine had a moderate or high migraine-related disability at MIDAS score [14], while in the one by Kister et al., 77% of pwMS had mild-to-moderate and 23% severe disability [7]. The monthly migraine days (MMD) were 7–15 days in 25.2% of the patients, >15 days in 12% of them, and continuous headache in 10% of them. Therefore, 47.2% of pwMS with migraine had at least 7 days/month of pain [14]. Comparing migraine with other types of headache in MS (tension type and post-interferon beta treatment), Villani et al. found that pwMS with migraine had a higher frequency of days with headache per month (mean of 9.1 vs. 3.3 of tension type of headache) and a greater level of migraine disability at HIT-6 scores than pwMS with other types of headache (mean of 51.6 vs. 37.7 of tension type of headache) [15].

Some studies have shown that migraine and MS may begin in the same period or that migraine may worsen after the MS diagnosis. Among 1113 pwMS interviewed between 1967 and 1987, 44 had been cases whose onset or subsequent relapses had been predicted by a migraine-type headache. Twenty-seven patients had no previous history of migraine, and 12 (44%) of them had a simultaneous onset of headache and MS [16]. After several decades, a study published in 2024 confirmed the association between headache and MS onset or a worsening of headache after MS diagnosis. Among 281 pwMS with headache, 134 presented a new-onset headache in close temporal relation to the onset of MS and/or within 4 weeks from MS treatment start. The remaining 147 patients had a preexisting headache that in 50.3% worsened after MS diagnosis or treatment start [17] (Table 1).

#### 3.1.2. Characteristics of MS

Migraine in pwMS might lead to increased body pain perception. An extensive study, conducted on 949 pwMS from 4 Canadian MS centers over a 2-year period, found that among 164 pwMS and migraine in comorbidity, migraine was associated with an increased likelihood of chronic pain [18]. Pain-related symptoms, such as Lhermitte’s sign, occipital and trigeminal neuralgia, facial pain, temporo-mandibular joint pain, spasms, and restless legs syndrome, were 2–5 times higher in pwMS with migraine than pwMS without migraine [7].

Furthermore, at the Perceived Deficits Questionnaire, pwMS with migraine have worse self-reported cognitive deficits compared to pwMS without migraine (OR = 1.04, 95% CI: 1.02–1.06) [10].

Migraine is also a comorbidity associated with an increased likelihood of developing chronic fatigue [7]. This finding is also confirmed by a direct comparison between the two populations: pwMS with migraine presented higher score of Fatigue Severity Scores than those without migraine (5.0 vs 3.6, *p* < 0.001) [7]. Despite these additional symptoms, the disability in pwMS with migraine, assessed by the Patient-Determined Disability Score (PDDS), was mild and similar compared to a group of pwMS without any type of headache [7].

Considering the MS phenotype, the relapsing-remitting (RR) course occurs more often among pwMS with migraine compared with pwMS without MS, in which the progressive forms of MS prevail (79 people with RRMS and migraine, 20 patients with SPMS and migraine, 6 patients with PPMS and migraine among 509 recruited pwMS) [19].

Using the 54-item MS-specific QoL questionnaire (MSQoL-54), Villani et al. investigated the impact of comorbid migraine on the quality of life (QoL) of 44 pwRR-MS with migraine compared to a sex/age-matched sample of pwMS without migraine. Although the physical and mental composite scores of the MS QoL-54 questionnaire were not different between the two groups, pwMS with migraine had worse scores in role limitation due to physical problems (*p* = 0.035), bodily pain (*p* = 0.030), and in health perception (*p* = 0.023) subscales. Additionally, the scores of these subscales correlated with the MIDAS (respectively, r = −0.43, *p* = 0.003; r = −0.51; *p* < 0.001; r = −0.38; *p* = 0.01). It is important to note that, in this study, no differences were found between the two groups regarding the educational level, MS duration and Expanded Disability Status Scale (EDSS), Fatigue Severity Scale, Beck Depression Inventory State and Trait Anxiety Inventory, or Toronto Alexithymia Scale. Therefore, differences in some domains of MS QoL-54 might not have been influenced by anxiety, depression, educational level, alexithymia, fatigue, and disability. Therefore, examining and managing migraine in pwMS could help to improve their QoL [20] (Table 1).

#### 3.1.3. Other Comorbidities

Considering other comorbidities occurring in the two diseases, pwMS with migraine reported history of depression more frequently than pwMS without migraine, with a higher PHQ-9 score (8.2 vs. 4.7, *p* < 0.001) [7].

PwMS with migraine also presented high levels of anxiety, with a higher score on the anxiety score (PHQ) than those without migraine (4.1 vs. 6.5, *p*< 0.001) [7].

Sleep problems are reported in both diseases, and in particular, pwMS with migraine compared to pwMS without headache (evaluated with the Epworth sleep scale score) complain of poorer quality of sleep (8.1 vs 5.6, *p* < 0.001), although the hours of sleep per night do not differ (6.8 vs 6.7, *p* value not significant) [7].

Another important comorbidity to consider is hypovitaminosis D. A study evaluated the vitamin D, vitamin D-binding protein, vitamin D receptor (VITDR), high-sensitivity C-reactive protein, superoxide dismutase (SOD), catalase (CAT), glutathione peroxidase (GSH-Px), total antioxidant status (TAS), total oxidant status, and oxidative stress index values in 50 RRMS patients with migraine, 50 RRMS patients without migraine, and 50 healthy volunteers. The authors found that pwMS with migraine had lower vitamin D and VITDR values compared with pwMS without migraine (respectively, *p* = 0.014, *p* < 0.001). In addition, the SOD, CAT, GSH-Px, and TAS values were lower in pwMS with migraine than in pwMS without migraine (all *p* < 0.001) [21] (Table 1).

**Table 1:** Clinical aspects in people with MS and migraine.

Characteristics of migraine in pwMS	Types of migraine			
	Migraine without aura		Migraine with aura	
	16.5% 17% 16.3% 14.3% 16%		3.9% 6.9% 0% 2% 10%	Doi et al. [11] Beckman et al. [12] D’Amico et al. [13] Möhrke et al. [8] Kister et al. [7]
			Type of auras in migraine:	
			Visual auras in 44.1% visual/sensory in 38.2%, Only sensory in 17.6%	
	Migraine-related disability			
	Fragoroso et al. [14] 68.3% moderate/high disability Kister et al. [7] 77% mild-to-moderate disability 23% severe disability			
	Frequency of attacks			
	Fragoroso et al. [14] 7–15 days/month in 25.2%, >15 days/month in 12%, continuous headache in 10% Villani et al. [15] Mean of 9.1 days per month			
	Onset in relation to the diagnosis of MS			
	Freedman et al. [16] simultaneous onset of migraine and MS in 44% of pwMS without previous history of migraine Abdel Naseer et al. [17] 47.6% new onset headache in close temporal relation to the onset of MS and/or within 4 weeks from MS treatment start 52.3% preexisting headache that in 50.3% worsened after MS diagnosis or treatment start			
Characteristics of MS in comorbility with migraine	Pain perception			
	− Migraine was associated with an increased likelihood of chronic pain [18] − Pain-related symptoms were 2–5 times higher in pwMS with migraine than pwMS without migraine [7]			
	MS-related disability			
	− Migraine is associated with an increased risk of chronic fatigue [18]. − Fatigue Severity Scores in pwMS with migraine > pwMS without migraine [7]. − Patient-Determined Disability Score was mild and similar compared to a group of pwMS without any type of headache [7].			
	Cognitive features			
	At the Perceived Deficits Questionnaire, pwMS with migraine have worse self-reported cognitive deficits compared to pwMS without migraine [10].			
	MS phenotype			
	The relapsing-remitting course > the progressive forms [19]			
	MS-related quality of life in pwMS and migraine			
	Compared to pwMS without migraine, pwMS with migraine had worse scores in role limitation due to physical problems, bodily pain and in health perception [20]			
Other comorbidities	Depression	PwMS with migraine reported history of depression more frequently than pwMS without migraine [7] PHQ-9 score for pwMS with migraine > for pwMS without migraine [7]		
	Anxiety	Anxiety score (at PHQ) for pwMS without migraine < for pwMS with migraine [7]		
	Sleep problems	Compared to pwMS without migraine, pwMS with migraine had poorer quality of sleep, without difference of hours of sleep per night [7]		
	↓vitamin D	PwMS with migraine had lower vitamin D, VITDR, OD, CAT, GSH-Px, and TAS values compared with pwMS without migraine [21]		

Legend: ↓ vitamin D: hypovitaminosis D.

### 3.2. Radiological Features in People with MS and Migraine

#### 3.2.1. Introduction: Characteristics of White Matter Lesions in MS and in Migraine

Hyperintense lesions on brain and spinal cord T2-weighted MRI represent one of the radiological hallmarks of MS. Lesions in MS are at least 3 mm in long axis and are in juxtacortical, cortical, periventricular (with the main axis perpendicular to the lateral ventricles), infratentorial, and spinal cord regions [22,23].

Although with different features, the presence of multiple T2-hyperintense white matter lesions (WMLs) on brain MRI scans is a well-known finding in migraine patients. In migraine, the lesions are focal, with round or slightly elongated shape, median size of 2.5 mm, and mostly detected in the periventricular and juxtacortical regions and in the posterior circulation territory [24]. In particular, in the latter case, 85% of WMLs in the posterior circulation territory are in the cerebellum [25,26]. Therefore, T2-hyperintense WMLs are common in migraine and MS; indeed, Liu et al. reported that a percentage ranging from 24.4 to 34.5 of headache patients may fulfill the radiological 2010 McDonald criteria for MS diagnosis [27].

#### 3.2.2. Radiological Features in People with MS and Migraine

Recently, Lapucci et al. [28] demonstrated that 2016 MAGNIMS criteria [29] had a higher specificity in differentiating patients with clinically isolated syndrome (CIS) from migraine patients (100% vs. 87%) but a predictably lower sensitivity (63% vs. 72%). In particular, compared with people with migraine with aura, people with CIS have a significantly higher average number and volume of T2-hyperintense WMLs (17.9 ± 16.9 vs. 6.2 ± 11.9 and 3.1 ± 4.2 vs. 0.3 ± 0.6 mL; *p* < 0.0001), both globally and particularly in the infratentorial, periventricular, and juxtacortical areas (*p* < 0.0001). Lesion probability maps and voxel-wise analysis of lesions distribution identified the periventricular regions as the area with the highest probability of detecting T2-hyperintense WMLs in CIS patients and with a statistically significant association with CIS diagnosis [28]. The authors suggested that the presence of at least three periventricular lesions could minimize the risk of misdiagnosis [28].

Even Kamson et al. confirmed that pwMS have a significantly higher mean number ((24.5 ± 19.3 vs. 18.4 ± 12.3) and volume (*p* = 0.0041) of T2-hyperintense WMLs, but pointed out that in the migraine group, more WMH lesions were found in the frontal lobe compared to the parietal (*p* = 0.0007), temporal (*p* < 0.0001), and occipital (*p* < 0.0001) lobes [30]. 

Concerning WML locations, another study comparing MRI from pwMS and migraine patients found periventricular lesions in all pwMS and 32% of people with migraine and juxtacortical lesions in all pwMS and 53% of people with migraine. The authors, using double inversion recovery (DIR) imaging, identified no cortical lesions in migraine patients, whereas these were present in 60% of pwMS. Therefore, DIR imaging may be useful in assessing focal cortical involvement in the differential diagnosis of brain lesions [31].

Evidence that most MS lesions are vein centered has led to the identification of the “central vessel sign” (CVS) as a method that may improve the ability to distinguish MS from other diseases. Using FLAIR* on 3T MRI, Solomon et al. demonstrated that the identification of the central vessel sign, particularly in the subcortical and deep white matter, helps differentiate MS from migraine [32]. Specifically, the study was conducted in 10 pwMS and 10 people with migraine with at least two hyperintense lesions ≥3 mm. The median percentage of lesions with the CVS in MS patients was 84% compared with 22% in people with migraine (*p* = 0.008). When analyzing individual brain areas, in subcortical and deep regions, the median percentage of lesions with the CVS was significantly higher in pwMS than in people with migraine (88% vs. 19%, *p* = 0.004) [32].

The CVS was also studied in a cohort of 120 pwMS to investigate comorbidities (small vessel disease and migraine) as risk factors for perivenular and non-perivenular lesions, applying the spherical mean technique (SMT) diffusion model [33]. In these patients, migraine resulted in an independent predictor of the presence of non-perivenular lesions. The authors concluded that SMT may help in differentiating perivenular lesions, characterized by higher inflammation, demyelination, and fiber disruption, from non-perivenular lesions. The development of new non-perivenular lesions, especially in the deep/subcortical WM, should be considered a “red flag” to investigate comorbidities, such as migraine, in these patients [33].

Sivakolundu et al. observed sequential blood-oxygen-level-dependent (BOLD) signal reductions from the core towards the perimeters in MS lesions, while not in lesions of non-specific white matter diseases (NSWMD) such as migraine and microvascular disease. BOLD slope, an indicator of lesion metabolic capacity, was significantly lower in MS compared to NSWM lesions (*p* = 0.0006), suggesting decreased metabolic activity in MS lesions [34].Recently, an MRI study with diffusion- and perfusion-weighted imaging and single-voxel proton magnetic resonance spectroscopy showed that migraine patients had the lowest intralesional creatine + phosphocreatine and myo-inositol (mI) values, while pwMS showed the highest intralesional T1 and T2 relaxation times, ADC, and mI values. No differences in perfusion variables were observed in any group. Therefore, despite some differences in advanced MRI measures, it is not currently possible to identify a clearly different pattern between the two WML patterns [35].

Even Orsi et al. compared the diffusion-weighted images between pwMS and people with migraine. They did not find significant differences for the high lesional ADCmono values (*p* = 0.2134) between groups. In migraine lesions, the biexponential measurements showed significantly higher ADCfast, ADCslow, and Pslow values than in MS lesions (*p* = 0.0344, *p* = 0.0019, *p* = 0.0021, respectively) [36].

In addition to the differential diagnosis of WMLs between MS and migraine, MRI studies have also investigated lesional areas associated with an increased risk of having migraine in pwMS. Interestingly, already in 1989, Freedman and Gray hypothesized the involvement of the midbrain region in migraine patients with MS. They found that 52% of patients, whose initial attack or subsequent MS exacerbations were heralded by migraine, had symptoms related to posterior fossa lesions [16]. In particular, pwMS with lesions within the midbrain/periaqueductal gray matter areas (PAG) had a four-fold increase in migraine-like headaches and a 2.7-fold increase in the combination of migraine and tension-type headaches when compared to pwMS without midbrain/PAG lesions [37]. In addition, another study, analyzing the frequency distribution and mean number of lesions in the different brainstem regions, found a significant involvement of the red nucleus and substantia nigra in pwMS with migraine, compared with pwMS without migraine. Therefore, compared with migraine patients, pwMS with migraine have a frequent involvement of the substantia nigra and PAG [38]. Recently, this association was not only confirmed, but it was also related to the frequency of attacks; in particular, lesions in the PAG were observed in 40% of pwMS with MMD > 10, 33.3% with MMD ranging from 3 to 10, and 26.7% with headaches occurring < 3 days per month [17].

The role of PAG in pwMS with migraine was also explored with functional MRI, identifying three specific patterns of PAG connectivity reorganization in pwMS with migraine [39]:

- Decreased positive resting state-functional connectivity (rs-FC) between the PAG and default mode network (DMN), the basal ganglia network (BGN), cerebellum, and left posterior caudal pons (the anatomical site of the trigeminal nucleus). The loss of PAG positive connectivity with the DMN and the left posterior cranial pons was associated with a high frequency of migraine attacks (evaluated with MMD).
- The loss of PAG negative connectivity with the sensorimotor and visual networks is linked to migraine symptom severity and related daily activities impact (evaluated with the visual analog scale (VAS-P) and the six-item Headache Impact Test (HIT-6).
- A PAG negative connection with the prefrontal executive control network, inversely correlated with MMD.

The occurrence of migraine, its severity, and related daily activities impact in pwMS were not substantially related to the T2-visible lesion load but were associated with changes in brain, and in particular PAG, connectivity networks.

Some studies evaluated the difference in lesion load among pwMS with and without migraine. Kister et al. found no differences in the number or distribution of T2 hyperintense lesions or the number of gadolinium-enhancing lesions in the two groups [7]. Conversely, Graziano et al. found that pwMS with migraine presented an increased number (*p* = 0.019) and volume (*p* = 0.022) of contrast-enhancing lesions than those without migraine [19] (Table 2).

### 3.3. Treatment

#### 3.3.1. Influence of MS-DMTs on Migraine

Headache is a known side effect of interferon beta (IFNβ-1) therapy, and it has been investigated in several studies. Worsening of pre-existing headaches or development of de novo headache occurred in pwMS treated with IFNβ-1 [17,40]. Primary headaches was more frequent in pwMS treated with IFNs (72%) compared to patients treated with other immunotherapies (azathioprine and glatiramer acetate) and in particular 34.2% of them had migraine without aura [41]. de Jong et al. found that pwMS with migraine (ORadj 1.55, 95%CI 1.18–2.04) were more likely to have previous exposure to IFN-b than controls [42].

The risk of headache during treatment with INF-β-1a 44 μg SC and IM was greater than with 22 μg of SC [43]. Only 6% of pwMS treated with glatiramer acetate reported headache [40]. 

Among a total of 63 pwMS with comorbid migraine, a longitudinal analysis showed a significant reduction of migraine frequency (from a mean value of 8.4 to 5.1 days per month; *p* = 0.034) and MIDAS score (from a mean value of 14.2 to 10.5; *p* = 0.045) in the subgroup patients switched from IFN β to Natalizumab (NTZ) [44].

According to the search criteria, no additional data on migraine and other DMTs were found, however, some meta-analysis evaluated the incidence of headache in randomized clinical trials (RCTs) enrolling pwMS, comparing B-cell-targeted therapies (rituximab, ocrelizumab, ofatumumab, ublituximab, or cladribine) with placebo. A trend in headache risk was found in the B-cell-targeted treatments, but it was not statistically significant. Sub-analyses found a significant association between cladribine and an increased incidence of headache, which the authors speculate to be related to the purinergic signaling cascade [45].

A network meta-analysis, comparing the safety profiles of high-efficacy DMTs, found that headache was more common for alemtuzumab as compared with all the other DMTs and placebo, as well as for cladribine versus natalizumab and for fingolimod versus natalizumab [46]. These results support data about cladribine in the aforementioned meta-analysis [45] but do not confirm the results of a meta-analysis by Lucchetta et al. [47]. They did not find a prevalence of headache in any DMT group compared to placebo. It must also be noted that, considering all side effects, the study showed a similar safety profile for DMTs and placebo [47] (Table 3).

#### 3.3.2. Influence of Migraine Treatments on MS

According to the search criteria, a single study specifically investigates the influence of migraine treatments on MS [48]. However, although not as the result of the this search, we will list the prophylactic treatments of migraine, and we will comment on the adverse effects that could influence MS symptoms.

Although the available treatment to manage migraine in MS is not different from that indicated in the general population, it is important to consider both adverse events on MS symptoms and multiple drug interactions.

Tricyclic antidepressants, prophylactic medications for migraine, could aggravate urinary retention or constipation, two frequent symptoms of MS. Moreover, tricyclic antidepressants could increase the risk of an irregular heart rhythm if administered with a sphingosine 1-phosphate receptors (S1P) modulator, such as fingolimod [49].

Other classes of prophylactic medications for migraine are beta blockers and calcium channel blockers. In these cases, it is necessary to consider the multiple drug interactions. In particular, treatment with a S1P modulator (such as fingolimod) should not be initiated in patients receiving these two classes of medications [50], for the risk of bradycardia.

Another treatment used in migraine prevention is topiramate; however, for this drug and the three previous pharmacological classes, studies demonstrating the efficacy and safety for the treatment of migraine specifically in pwMS are lacking.

Botulinum toxin type A (BoNT-A), indicated for the treatment of chronic migraine in the general population, is used to treat spasticity and neurogenic detrusor overactivity incontinence in MS. Therefore, its administration in MS is known [51] and largely used.

Eftekhari et al. demonstrated that BoNT-A was a safe and effective treatment for intractable chronic migraine in pwMS, decreasing weekly migraine days (from 6.28 to 1.16), pain intensity (from 94.8% to 26%), and duration (from 10.36 to 2.24 h) [52].

Monoclonal antibodies and gepants targeting the calcitonin gene-related peptide (CGRP) are a new milestone in migraine prevention.

According to the search criteria, the only study reporting the influence of anti-CGRP treatments on MS is that one by Gonzalez-Martinez et al. that demonstrated, in nine pwMS and comorbid migraine co-treated with DMTs, that the anti-CGRP treatment was generally safe and well tolerated [48]. Anti-CGRP therapies were erenumab, galcanezumab, or ubrogepant, and simultaneous DMTs were glatiramer acetate, fingolimod, dimethyl fumarate, teriflunomide or anti-CD20 therapy. There were no serious adverse events in the observational period. The main adverse events were infections (33%, urinary tract infection, sinus infection, and upper respiratory infection), injection site reaction (11%), and constipation (11%). However, none of these adverse events was severe enough to require stopping the anti-CGRP or DMT medication. In one patient, after three months, a 0.5-point increase in EDSS score was reported, and another one had a new lesion on brain MRI during the follow-up period [48]. In a recent study, Mason et al. confirmed the favorable efficacy and adverse event profile of anti-CGRP treatment in 27 pwMS treated with several DMTs [47]. The CGRP monoclonal antibodies were fremanezumab, eptinezumab, erenumab, or galcanezumab. The duration of therapy with CGRP monoclonal antibody was 4–12 months for 25.9% of patients, 13–23 months for 37.0% of them, 24–35 months for 18.5%, and >36 months for 18.5%. DMTs for MS were ocrelizumab, glatiramer acetate, siponimod, cladribine, natalizumab, fingolimod, interferon beta-1a, alemtuzumab, dimethyl fumarate, or teriflunomide. No worsening of MS was observed during the cotreatment period. Approximately 92.6% of participants reported a reduction in headache frequency (70.4% of those described a >50% reduction in migraine frequency). Mild AEs (i.e., muscle spasms, constipation, headache) were reported only in 11% of patients [53].

Moreover, migraine treatment is moving closer and closer to complementary and integrative health strategies, such as cognitive behavioral therapy. A study used smartphone-based behavioral therapy for 90 days to manage migraine pain in pwMS [54]. Sixty-six percent of patients reported significant satisfaction with this intervention, and 87% would recommend it to others at each follow-up visit. It is important to note that most patients were already on preventive medication for migraine (47% with oral medications and 15% with botulinum toxin) and had comorbidities (such as depression in 56% and anxiety in 52%) [54] (Table 3).

**Table 3:** Influence of MS-DMTs on migraine and of migraine treatments on MS. Legend: IFNβ-1: interferon beta 1; sc: subcutaneous; im: intramuscular; S1P: sphingosine 1-phosphate; BoNT-A: Botulinum Toxin type A; CGRP: calcitonin gene-related peptide. * studies that met the search criteria.

Influence of MS-DMTs on migraine	INF-β-1a *: Worsening of pre-existing headaches or development of de novo headache in INF-β-1a 44 μg sc and im > INF-β-1a 22 μg sc [40,41,42,43] and a significant reduction of migraine frequency and MIDAS score in switching from IFN β to natalizumab [44] Cladribine: was associated to increased incidence of headache [45] also when compared to natalizumab [46] Alemtuzumab: headache was more common for alemtuzumab as compared with all the other DMTs and placebo [46] Fingolimod: headache was more common when compared to natalizumab [46] No prevalence of headache in DMT-group compared to placebo [47]
Influence of migraine treatments on MS	Tricyclic antidepressants: no data specifically in pwMS with migraine, possible AE: aggravate urinary retention or constipation and increase the risk of an irregular heart rhythm with S1P modulators [49]. Beta blockers and calcium channel blockers: no data specifically in pwMS with migraine, possible AE: increase the risk of bradycardia with S1P modulators [50] Topiramate: no data specifically in pwMS with migraine BoNT-A: is safe and effective treatment for intractable chronic migraine in pwMS [52] Anti-CGRP therapies: favorable efficacy and adverse event profile [48] *

## 4. Discussion

Migraine is the most prevalent type of headache in pwMS, and migraine without aura is the most frequent form. In studies that differentiated types of migraine among pwMS, the percentage of patients experiencing migraine with aura was different and ranged from 0 to 6.9%. Instead, the prevalence of migraine without aura was similar across studies (with rates of 16.3% vs. 16.5% vs. 17%, and only one study reporting a slightly lower percentage, 14.3%) [4,7,8,11,12,13].

Migraine and MS may start at the same time; furthermore, migraine may worsen after the MS diagnosis. Moreover, migraine in pwMS has a high monthly frequency and migraine-related disability. Approximately 47.2% of pwMS with migraine had at least 7 days/month of pain, with a mean of 9.1 days/month [14,15]. Also compared with other types of headache in MS, migraine-related disability at HIT-6 is greater [15].

In contrast, MS-related perceived disability (at PDDS) in pwMS with migraine is not greater than in pwMS without migraine, although the clinical course is more symptomatic (mainly related to increased perception of body pain, fatigue, and difficulty in cognitive performance) [7]. This aspect greatly impacts the QoL of pwMS with migraine, who present worse scores, especially at the bodily pain (both at MSQoL-54 and SF-36) and social functioning (at SF36) subscales [8,15].

These data are important because, in addition to offering stimuli for further studies, they prompt us not to underestimate migraine pain in pwMS, as it is itself a cause of disability and has a negative impact on QoL. Furthermore, the average MMD and migraine-related disability should drive toward a preventive treatment, which should also consider the increased perception of bodily pain in these patients.

Regarding comorbidities, it is important to screen pwMS and migraine for depression, as it is common in both MS [55] and in migraine [56] patients. Interestingly, a study, conducted during the COVID-19 pandemic period, found that a higher percentage of people with migraine were depressed, compared to people with MS (50% vs. 43%, *p* = 0.04) [57]. Instead, pwMS with migraine had higher levels of depression compared with pwMS without migraine. PwMS with migraine also had higher levels of anxiety and worse sleep quality, compared with pwMS without migraine. Therefore, comorbidity between MS and migraine could expose patients to the risk of these mood disorders. Studies evaluating the efficacy of depression, anxiety, and insomnia treatment on both migraine features and some MS symptoms, such as cognitive symptoms or fatigue, would be interesting and could suggest further integrated therapeutic possibilities.

Hypovitaminosis D is another common comorbidity in both MS [58] and migraine [59] patients. Comorbidity between MS and migraine could increase the risk of finding not only low values of vitamin D and VITDR but also a change in oxidative stress markers (such as SOD, CAT, GSH-Px, and TAS) with respect to MS patients without migraine [21].

These results are interesting, considering the possible advantages of vitamin D supplementation for its anti-inflammatory effect both in MS [58] and in migraine [60] and for its possible impact on improving migraine characteristics [60]. These data could stimulate studies that aim to evaluate the efficacy of vitamin D supplementation on both migraine features and the course of MS in patients in whom these diseases coexist.

Migraine and MS also share some radiological features, which can lead to diagnostic challenges. Several studies investigated differences in MRI patterns in MS and migraine. Lesions in MS are at least 3 mm in long axis [23], while in migraine the lesions are focal, with round or slightly elongated shape, median size of 2.5 mm [24]. Concerning WML location, although some areas are in common between migraine and MS, pwMS have a significantly higher average number and volume of T2-hyperintense WMLs [28]. Moreover, the presence of cortical lesions in DIR imaging [31] or at least three periventricular lesions in T2-MRI [28] or perivenular lesions (with SMT diffusion model) [33] may point toward MS lesions. Using FLAIR* on 3T MRI, the identification of the CVS, particularly in the subcortical and deep white matter, helps differentiate MS from migraine [32]. MRI represents a useful tool both for the differential diagnosis of WMLs in MS and migraine and in the follow-up of MS patients with migraine. Recently, the new diagnostic criteria for MS have been presented at ECTRIMS 2024 [61]. It is conceivable that they may reduce misdiagnosis by improving MRI protocols (by introducing CVS and paramagnetic rim lesions, PRL), focusing on optic nerve diagnosis studies, and implementing CSF analysis [61]. Also, in the presentation of the new diagnostic criteria, the need for more stringent features to confirm the diagnosis of MS in individuals with migraine emerged. In particular, in patients with suspected MS presenting with headache disorders, including migraine, additional features are strongly recommended to confirm or not the diagnosis of MS: spinal cord lesions, six CVS lesions, and positive CSF.

Furthermore, MRI studies have investigated the pathogenetic link between MS and migraine, which might be related, in some cases, also to the WML location, disrupting the pathways involved in migraine pathogenesis. Indeed, pwMS with lesions within the midbrain/PAG had a four-fold increase in migraine compared to pwMS without midbrain/PAG lesions [37]. Therefore, compared with migraine patients, pwMS with migraine have a frequent involvement of the substantia nigra and PAG [38]. This finding underlined the role of the red nucleus, substantia nigra, and PAG in pain modulation, and particularly in migraine pathogenesis.

A functional MRI-BOLD study [62] demonstrated that in 75% of people without MS who developed migraine (with and without aura) during a repetitive visual stimulation, the baseline T2*-weighted MR signal intensities increased in the red nucleus and substantia nigra, suggesting that these brainstem structures are part of a neuronal network activated during an attack. It is worth reminding that the substantia nigra is linked to the PAG, which is the inhibitor center of afferent trigeminal nociceptive traffic [63].

In pwMS, migraine severity and the impact on daily activities are more associated with changes in brain connectivity networks than with T2-visible lesion load. In particular, a change in the connectivity pathway between PAG and the left posterior caudal pons, the anatomical site of the trigeminal nucleus sensorimotor and visual network (the association between migraine and increased excitability in the sensorimotor cortex is well known [64]), and the prefrontal cortex, considered the main source of cognitive control over pain perception.

In particular, the latter changes could be linked to the increased prevalence of pain symptoms (such as Lhermitte’s sign, occipital and trigeminal neuralgia, facial pain, temporomandibular joint pain, spasms, and restless legs syndrome) in pwMS with migraine compared to pwMS without migraine [7].

Interestingly, cortical demyelination is associated with an acceleration of cortical spreading depression in rodent models, assuming that the involvement of cortical myelin in the stabilization and buffering of extracellular ion content is decisive for propagation velocity and cortical excitability, respectively [65].

All these clinical and radiological features of comorbidity between the two diseases should be considered when planning a treatment strategy.

Comparing the safety profiles of MS-DMTs, headache is surely a known side effect of IFNβ-1 therapy [40,41,42,43], but some metanalysis reported that it was more common also for alemtuzumab as compared with all the other DMTs and placebo and for cladribine versus natalizumab and for fingolimod versus natalizumab, although the prevalence of headache in some high-efficacy DMTs has not been confirmed by other studies [45,46,47].

According to the criteria, a single study [48] emerges from the literature review that specifically investigates the influence of migraine treatments on MS. However, the authors will still list the drugs used in the prevention of migraine, and possible adverse effects in this case will only be reported in relation to the general known adverse events or other available information. The prophylactic medications for migraine can be helpful in treating pwMS with migraine; however, they should be administrated considering both the adverse events on MS symptoms and multiple drug interactions, in particular tricyclic antidepressants, beta blockers, calcium channel blockers, and S1P modulators.

It has been demonstrated that fingolimod inhibits acid sphingomyelinase by a mechanism similar to tricyclic antidepressants [66]. However, amitriptyline could also have positive effects on microglia, as demonstrated by in vitro studies: in particular, a significant reduction in the inflammation extension, with the inhibition of microglial activation, was observed in the incubation of inflammatory astrocyte/microglia co-cultures with amitriptyline, doxepin, or IFN-β alone, or co-incubation of IFN-β pre-treated co-cultures with both antidepressants [67].

It should also be considered that the use of tricyclic antidepressants could have an impact on cognitive performance; this should be carefully considered, given the possible decline in cognitive functions in pwMS [68]. However, studies specifically demonstrating the effect of tricyclics on cognitive functions in pwMS are needed to confirm these effects during migraine treatment.

Regarding beta blockers, it is interesting to note the effect of propranolol (the beta blocker most used in migraine) in a murine model of experimental autoimmune encephalomyelitis (EAE). Propranolol decreased the incidence of EAE and prolonged the duration of the asymptomatic disease phase due to a reduction in migration of CD11b+ antigen-presenting cells and of activation and proliferation of CD4+ T cells in lymph nodes, as well as a decreased number of Th17 cells in lymph nodes and spinal cord [69]. The effect of propranolol in EAE was also demonstrated by enhancing immunoregulatory/protective properties of murine spinal cord microglia due to upregulation of CX3CR1 expression [70].

Regarding topiramate, studies demonstrating the efficacy and safety of this drug for the treatment of migraine specifically in pwMS are lacking; however, it is relevant to consider the adverse events reported during treatment with topiramate, such as paraesthesias and cognitive disorders, overlapping problems with MS symptoms [71].

BoNT-A is used in MS not only for the treatment of chronic migraine but also for spasticity and neurogenic detrusor overactivity incontinence [51]. Eftekhari et al. demonstrated that BoNT-A was a safe and effective treatment for intractable chronic migraine in pwMS [52]; however, other studies specifically demonstrating the efficacy and safety of BoNT-A for the treatment of chronic migraine in MS are needed. Therefore, it is crucial to invest in studies that explore the potential benefits of BoNT-A in patients with MS and chronic migraine, also to provide valuable insights into the definition of targeted therapy schedule.

**Figure 2:**
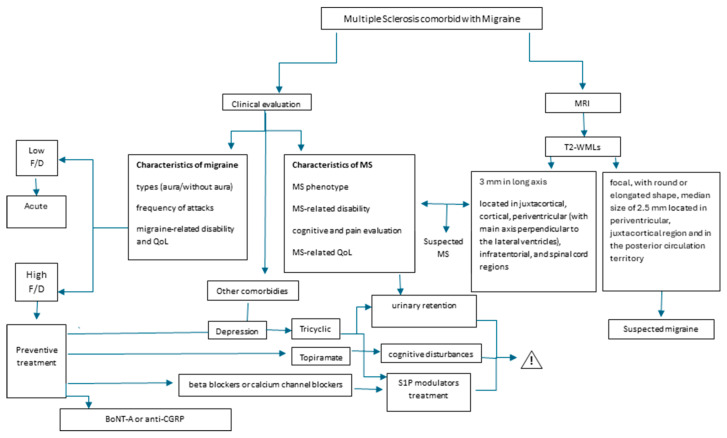
Flow chat clinical and radiological evaluation in multiple sclerosis comorbid with migraine. Legend: F/D: frequency or/and disability; BoNT-A: botulinum toxin type A; anti-CGRP: anti-CGRP drugs; T2-WMLs: T2-hyperintense white matter lesions.

Concerning the anti-CGRP treatment in MS patients co-treated with MS-DMTs, the available studies demonstrated the favorable efficacy and adverse event profile [48,53]. However, the studies had small sample sizes, and the median follow-up of simultaneous therapies varied from 9–13.5 months (depending on the anti-CGRP monoclonal antibody) [48] to 15 months [53]. Therefore, further studies are necessary to extend the sample size and follow-up. Figure 2 shows a concluding schematic evaluation of the clinical and radiological assessment in multiple sclerosis in comorbidity with migraine.

In addition to the scarcity of data on sample size and follow-up, the lack of randomized clinical studies on migraine prevention drugs in combination with DMTs could represent a limitation to a comprehensive evaluation. A further limitation is the scarcity of clinical and radiological studies that comprehensively evaluate the course of the two diseases over time, the clinical–radiological correlation, and studies analyzing the clinical-radiological features during a migraine attack in patients with MS. Another limitation is the exclusion of articles in languages other than English, as they might have added to the review.

Moreover, other studies should move closer and closer to complementary and integrative health strategies, also associated with technology, such as cognitive behavioral therapy on smartphones, toward which most of pwMS and migraine have shown interest.

## 5. Conclusions

Migraine and MS can frequently present as comorbidities but can also influence each other, with a negative impact on the QoL of pwMS with migraine. In the future, the treatment of migraine in MS should include an increasingly multipurpose approach, aiming at the management of fatigue, depression, anxiety, insomnia, and increased pain perception.

Further research is needed to clarify the nature of this connection and to determine if effective management of one condition could potentially impact the other. Nonetheless, this finding highlights the importance of screening pwMS for migraine and providing appropriate treatment to optimize their health and QoL. Furthermore, considering the global burden of individual diseases as causes of disability worldwide, it would be interesting to assess the ranking of MS-migraine comorbidity in the global disease burden.

## Figures and Tables

**Table 2:** Radiological aspects in people with MS and migraine. Legend: pwMS: people with MS; PAG: periaqueductal gray matter areas; rs-FC: resting state-functional connectivity; DMN: default mode network; ↓: decrease.

White matter lesions	MS pattern [22,23,32,33]	at least 3 mm in long axis juxtacortical, cortical, periventricular (with main axis perpendicular to the lateral ventricles), infratentorial, and spinal cord regions central vessel sign
	Migraine pattern [24,25,26]	focal, with round or slightly elongated shape, median size of 2.5 mm periventricular, juxtacortical and posterior circulation territory (cerebellum) no cortical
	Risk lesions of migraine in pwMS [17,37,38]	midbrain/PAG matter areas red nucleus and substantia nigra
f-MRI connectivity [39]		↓ rs-FC between the PAG and DMN, the basal ganglia network, cerebellum, and left posterior caudal pons loss of PAG negative connectivity with sensorimotor and visual network a PAG negative connection with the prefrontal executive control network
